# Processes in arithmetic strategy selection: a fMRI study

**DOI:** 10.3389/fpsyg.2015.00061

**Published:** 2015-02-04

**Authors:** Julien Taillan, Eléonore Ardiale, Jean-Luc Anton, Bruno Nazarian, Olivier Félician, Patrick Lemaire

**Affiliations:** ^1^Laboratoire de Psychologie Cognitive, Centre National de la Recherche Scientifique and Aix-Marseille UniversitéMarseille, France; ^2^Centre d’IRM Fonctionnelle Cérébrale de Marseille, CHU Timone, INT – UMR 7289Marseille, France; ^3^Aix Marseille Université, INS UMR_S 1106, 13005Marseille, France; ^4^APHM, CHU Timone, Service de Neurologie et NeuropsychologieMarseille, France

**Keywords:** strategy selection, arithmetic problem solving, fMRI, anterior cingulate cortex, dorso-lateral prefrontal cortex

## Abstract

This neuroimaging (functional magnetic resonance imaging) study investigated neural correlates of strategy selection. Young adults performed an arithmetic task in two different conditions. In both conditions, participants had to provide estimates of two-digit multiplication problems like 54 × 78. In the choice condition, participants had to select the better of two available rounding strategies, rounding-up (RU) strategy (i.e., doing 60 × 80 = 4,800) or rounding-down (RD) strategy (i.e., doing 50 × 70 = 3,500 to estimate product of 54 × 78). In the no-choice condition, participants did not have to select strategy on each problem but were told which strategy to use; they executed RU and RD strategies each on a series of problems. Participants also had a control task (i.e., providing correct products of multiplication problems like 40 × 50). Brain activations and performance were analyzed as a function of these conditions. Participants were able to frequently choose the better strategy in the choice condition; they were also slower when they executed the difficult RU than the easier RD. Neuroimaging data showed greater brain activations in right anterior cingulate cortex (ACC), dorso-lateral prefrontal cortex (DLPFC), and angular gyrus (ANG), when selecting (relative to executing) the better strategy on each problem. Moreover, RU was associated with more parietal cortex activation than RD. These results suggest an important role of fronto-parietal network in strategy selection and have important implications for our further understanding and modeling cognitive processes underlying strategy selection.

## INTRODUCTION

During the last 30 years, research on human cognition has shown that, in many domains, people use several strategies to accomplish cognitive tasks (Siegler, 2007). A strategy can be defined as “a procedure or a set of procedures for achieving a higher level goal or task” (Lemaire and Reder, 1999, p. 365). Strategy variability enables participants to alternate flexibly between different strategies from one problem to the next so as to adapt to the demands of problems and situations. One of the crucial issues concerns how we choose a strategy among several available strategies to solve each problem. Although a number of behavioral studies have investigated strategy selection processes, no previous works examined the neural bases of strategy selection, which we do here in a functional magnetic resonance imaging (fMRI) study. We first review previous relevant findings on strategy selection from behavioral studies and on brain activations in frontal and prefrontal lobes for tasks that require executive functions involved in strategy selection.

Behavioral studies have shown that participants select strategies on a trial-by-trial basis (i.e., they choose among available strategies on each problem) and that performance depends on the strategies they use, how often they use each available strategy, how fast and accurate they are when they execute each strategy, and how they select strategies on each problem. Previous studies have also found that participants’ strategy choices are influenced by several parameters like characteristics of problems (e.g., participants retrieve solutions directly in memory more often on small arithmetic problems like 3 × 4 and use counting more often on large problems like 7 × 8; Zbrodoff and Logan, 2005), of situations (e.g., participants tend to use retrieval strategy more often under speed pressures when solving arithmetic problems; Campbell and Austin, 2002), of strategies (e.g., participants are faster when they use memory retrieval than when using counting strategies to solve arithmetic problems; Imbo and Vandierendonck, 2007), and of participants (e.g., as children grow older they use retrieval strategy more and more often; Lemaire and Siegler, 1995). Over and above main effects, all these characteristics interact when participants select strategies on each problem. For example, Lemaire et al. (2004) found that in a computational estimation task, participants chose a rounding-down (RD) strategy more often on small-unit problems (e.g., 62 × 47 = 60 × 40 = 2400) than on large-unit problems (e.g., 78 × 24 = 70 × 20 = 1400). Moreover, this difference was larger in younger than in older adults; and such age differences increased when accuracy was emphasized.

Several computational models of strategy selection have attempted to formalize the mechanisms by which people choose strategies on each problem: Lovett and Anderson’s (1996) ACT–R; Lovett and Schunn’s (1999) RCCL; Payne et al. (1993) adaptive decision maker; Reder and Ritter’s (1992) model; Rieskamp and Otto’s (2006) SSL model; Schunn et al. (1997) SAC model; and Siegler and Araya’s (2005) SCADS^∗^. Previous works by Reder and Ritter (1992) and Schunn et al. (1997) has shown that participants can base strategy selection on a rapid feeling of knowing (FOK) process. Consistent with this view, Reder and Ritter (1992) found that participants’ FOK judgments were predicted by the familiarity with components of the problem. Other models, like RCCL, SSL, and SCADS^∗^, argued that strategy selection processes involve associative mechanisms, like activating relative costs/benefits of each strategy. These mechanisms are more complex and require more processes, such as the analysis of the problems’ characteristics. All models also assume that strategies including fewer and/or simpler procedures (e.g., retrieving correct solution of arithmetic problems like 3 × 4 directly from memory) are easier to execute than strategies including more and/or more complex procedures (e.g., adding 3 four times). These assumptions proved sufficient to account for most findings on strategy choices and strategy execution.

However, an important limitation of previous works concerns the limited understanding of the fact that participants do not always choose the better strategy on each problem. For example, sometimes people repeat the same strategy over two consecutive trials, whereas using a different strategy on the second problem would have yielded better performance (e.g., Luwel et al., 2009; Lemaire and Lecacheur, 2010). Such findings suggest that strategy selection involves additional mechanisms not originally assumed by previous theoretical models. Several recent findings are consistent with this possibility. For example, Hodzik and Lemaire (2011) found that better strategy selection correlated with executive functions. Such findings suggest that selecting a strategy on a given problem is not just a product of considering problem and strategy characteristics as assumed by models of strategy selection. Other mechanisms, like executive control mechanisms for example, are crucial for strategy selection. Neuroimaging approach was adopted here with the hope that brain activations would fruitfully help to precise mechanisms involved in strategy selection. For example, discovering that better strategy selection is accompanied by brain activations in frontal and prefrontal areas would be additional pieces of evidence that strategy selection involves executive control processes and would prompt revisions of theories of strategy selection.

## OVERVIEW OF THE PRESENT STUDY

In this study, our goal was to examine the neural activations associated with strategy selection. We pursued this goal in the context of arithmetic problem solving tasks as previous research found that this is a good context to investigate strategic aspects of human cognition and that findings in this context generalize to other cognitive domains (e.g., Lemaire, 2010). Participants performed a computational estimation task in which they were asked to select (and execute) the better of two available strategies. They had to provide the better estimate of multiplication problems (e.g., 36 × 52) while using either the rounding-up (RU) strategy (40 × 60 = 2,400) or the RD strategy (30 × 50 = 1,500). Following the choice/no-choice method proposed by Siegler and Lemaire (1997), we tested participants in two different conditions, a choice condition, and a no-choice condition. In the choice condition, participants had to select the better strategy on each problem and execute it. Participants were explained that the better strategy is the strategy that, among the available RD and RU strategies, enables to provide the product that is the closest product from the correct product. Participants were not informed about whether there is a rule to determine which strategy is the better strategy on each problem. In the no-choice condition, participants were asked to execute strategies without selecting strategies on each item. That is, they were asked to use a pre-specified strategy on each problem. Brain activations were compared between choice and no-choice condition to dissociate brain activations associated with strategy execution from those associated with strategy selection.

Two sets of predictions were tested, one each on behavioral performance and brain-imaging data. Regarding participants’ performance, we expected to replicate previous findings in computational estimation tasks (e.g., Lemaire et al., 2000, 2004; Lemaire and Lecacheur, 2010). Participants should select each strategy when it works better. That is, they should use the RU strategy more often on RU problems (e.g., doing 40 × 60 for 37 × 58) and the RD strategy on RD problems (e.g., doing 30 × 50 for 32 × 54). They should be slower and less accurate when they choose and execute the RU strategy than the RD strategy, as rounding up is harder than rounding down. As analyzed in previous studies, this comes from the fact that, when participants use the RU strategy, they have to increment both operands up to the nearest larger decades whereas when using the RD strategy they can use decades that are still displayed on the screen.

Regarding brain activations, as strategy selection involves a component choice (i.e., choosing between two rounding strategies), we expected specific activations in ACC to the extent that ACC is crucial in choice behaviors (e.g., Anderson et al., 2009b; D’Esposito et al., 2009). Moreover, to select a strategy, participants should maintain active both rounding strategies in memory and flexibly alternate between these two strategies. Such maintenance and strategy switching are assumed to rely on executive control processes. Therefore, following previous findings of frontal and prefrontal activations in tasks requiring executive control processes (e.g., Dove et al., 1999), we also expected activations in prefrontal cortex. This would support behavioral findings regarding the crucial role of executive functions during strategy selection.

With regards to brain activations underlying strategy execution, given the lack of research on neural bases of strategy execution, it was hard to make specific and precise predictions. Nevertheless, a previous research by Gandini et al. (2008) found that when participants used two different strategies to accomplish numerosity estimation tasks, each strategy was sustained by both overlapping and distinct brain areas. This may not be limited to numerosity estimation tasks; it may also generalize to arithmetic problem solving tasks like computational estimation tasks tested here. This is highly likely as the two computational estimation strategies tested here yielded different levels of performance in previous works (e.g., Lemaire et al., 2000, 2004; Lemaire and Lecacheur, 2010; Uittenhove and Lemaire, 2012, 2013; Uittenhove et al., 2013).

Most of previous studies investigating the neural bases of mathematical cognition found activations in the parietal regions when people accomplish arithmetic tasks (e.g., Piazza et al., 2004; Pinel et al., 2004; Castelli et al., 2006). More specifically, solving complex arithmetic problems was mainly associated with increased activations in left and right posterior lobes, and in left frontal cortex (e.g., Zago et al., 2001; Grabner et al., 2007). Moreover, as solving complex arithmetic problems involves mental processes like visual processing, manipulation, and storage of numbers in working-memory, we expected activations of brain areas usually activated by these processes. Indeed, brain activations were observed in superior frontal sulcus when solving visuo-spatial working-memory tasks (e.g., Klingberg, 2006), in supramarginal gyrus (SMG), and in occipito-temporal junction when participants maintained and manipulated items or intermediate results in working-memory (e.g., Petit and Zago, 2002). Therefore, we expected larger activations in left and right posterior lobes (parietal and occipital lobes), as well as in frontal lobe when participants execute the RU strategy than when executing the RD strategy.

## MATERIALS AND METHODS

### PARTICIPANTS

Twenty-two right-handed participants were tested. They were undergraduate students from Aix-Marseille University, and their first language was French. Participants were excluded if they were taking any CNS-active or vasoactive medication, or if they reported significant neurological, cardiovascular, psychiatric, or systemic illness. Prior to participation, all participants signed information consent, and were informed that they could withdraw from the study at any time. Four participants were excluded from this study because they did not perform the task well. Two participants chose the better strategy on 50% of problems, which did not significantly differ from a random selection, and the other two participants used the RD strategy on all problems. Data from a sample of 18 participants (nine men) from the age range 19–30 (mean 24.0 years) were analyzed.

For purpose of comparisons with previous studies and in order to test participants with a minimum level of arithmetic fluency, we assessed participants’ arithmetic skills with an independent, paper-and-pencil test, the so-called French Kit test (French et al., 1963). This test consists in subtests of addition, subtraction, and multiplication problems. Each subtest contains two pages of problems. The addition subtest consists in two pages of addition problems presented in columnar (e.g., 47 + 63 + 9), and two pages of subtraction/multiplication subtest which consists in alternation between a row of subtraction problems and a row of multiplication problems. All participants were told that they have 2 min to accurately solve as many problems as possible on one page. The correctly solved problems for each page were summed to yield a total arithmetic score. As participants are supposed to give their answer within a limited time, only participants with an arithmetic score of at least 60 were included in the study (mean 81). This was done in order to test only participants with a good level in arithmetic.

### STIMULI

We tested two types of experimental problems. Half the problems were so-called small-unit problems and the other large-unit problems. Small- and large-unit problems differed on the basis of the size of the sum of unit digits. Sums of unit digits were smaller or equal to 10 (7–10) and larger or equal to 10 (10–13) for small- and large-unit problems, respectively. Also, small- and large-unit problems differed on relative strategy accuracy. Relative strategy accuracy for a given problem was based on mean percent deviations between estimates and correct products, calculated with the following formula: |[(estimate product – correct product)/correct product] × 100|. For each problem we calculated percent deviation between correct product and estimate. For example, percent deviations were 26.9 and 16.9% for 38 × 54 when using RD and RU strategies, respectively. For this problem, the better strategy was the RU strategy. Absolute differences in mean percent deviations between correct products and estimates for small-unit problems were 15.1% (range = 8.3%-24.8%) and 21.0% (range = 13.8%-28.8%) when using the RD and RU strategies, respectively. Similarly, mean percent deviations between correct products and estimates for large-unit problems were 23.1% (range = 13.1%-40.3%) and 15.2% (range = 8.9%-22.1%) when using the RD and RU strategies, respectively. Small- and large-unit problems had comparable correct products when solved with each rounding strategy. More precisely, mean correct products were 3,330 (range = 1,426-6,364) and 3,355 (range = 1,638-5,568) for small- and large-unit problems, respectively. This was necessary for determining which strategy is the better on each problem to be unconfounded with the difficulty of calculating the correct product.

Given effects that are known in the domain of mental arithmetic (see Campbell, 2005, for an overview), when selecting the experimental problems, we controlled the following factors: (a) no operands had 0 (e.g., 30 × 48) or 5 as unit digit (e.g., 35 × 48); (b) the decades were between 3 and 8; (c) digits were not repeated in the same decade or unit positions across operands (e.g., 43 × 47); (d) no digits were repeated within operands (e.g., 44 × 59); (e) no reverse order of operands were used (e.g., if 52 × 76 was used, then 76 × 52 was not used); (f) no tie problems (e.g., 32 × 32) were used; (g) the first operand was larger than the second in half the problems; (h) no operands had its nearest decade equal to 0, 10, or 100. Control problems were constructed such that (a) both operands had its unit digits equal to 0, (b) the decades were between 3 and 9, and (c) no decades were equal (e.g., 40 × 40).

We created 120 experimental problems and 60 control problems. In the choice condition, each of the two sets contained 60 experimental and 60 control problems. The same experimental and control problems used in the choice condition were mixed and presented into two sets of problems in the no-choice condition. These four sets were equal for mean correct products and mean percent deviations between correct products and estimates, for experimental problems, and for mean correct products for control problems.

### PROCEDURE

Participants had a training session one to 2 weeks before entering the fMRI scanner. This was to familiarize them with the task environment (see **Table 1**). The RD strategy was described as rounding both operands down to the nearest smaller decades, like when doing 50 × 80 to estimate 51 × 89. The RU strategy was described as rounding both operands up to the nearest larger decades, like when doing 60 × 90 to estimate 51 × 89. Then, participants had to choose which one of these two rounding strategies yields the better estimate. Instructions emphasized that participants should not use any other strategies, should do only the initial rounding up or down, and should do nothing more (i.e., adding or subtracting small amounts) after calculating the product of rounded operands.

**Table 1:** Summary of the procedure.

Session	Task	Condition	Number of trials
Session 1	Standardized tests	French Kit test	As many as possible
		Mill Hill test	33
	Familiarization with the computational estimation task	Rounding-down (RD) condition	20
		Rounding-up (RU) condition	20
		Choice condition	40
	Practice to the experimental task: sequence of three problems	Choice condition	12
		RD condition	12
		RU condition	12
Session 2	Practice to the experimental task: sequence of five problems	Choice condition	40
		RD condition	20
		RU condition	20

After a few minutes rest, participants practiced the experimental task. Participants were told to pay attention to the symbol above the multiplication problem. An arrow pointing up “↑”cued participants to use the RU strategy; an arrow pointing down “↓”cued participants to use the RD strategy; a question mark “?” cued participants to choose among the two rounding strategies which one yields the better estimate, and the sign “=” cued them to calculate the exact answer. The latter symbol was used only for control multiplication problems.

Participants were told that they were going to encounter three different conditions as in the previous practice. They were first tested in the choice condition (i.e., choosing the better rounding strategy) and then in the no-choice RD and RU conditions. These latter two sets were counterbalanced between participants. Based on previous works (e.g., Siegler and Lemaire, 1997), we decided to test participants in the choice condition first as this order enables their strategy choices to not be contaminated by recent execution of strategies in the no-choice condition when no-choice conditions are taken before the choice condition. Participants were informed that multiplication problems were displayed with different durations. Multiplication problems cued with “?” were displayed during 7.5 s, problems cued with “↑” during 4 s, problems cued with “↓,” and problems cued with “=” during 3.5 s. Lengths of trials differed because participants needed more time in the choice condition to choose between the two rounding strategies. Likewise, in the execution condition, RD problems are known to be solved more quickly than RU problems because they are easier. Participants were instructed to answer before the multiplication problems disappeared. They gave their estimates aloud. For example, for “↓” 31 × 57, participant should say “1,500” and then click as fast as possible on the mouse button. Verbal protocols and solution latencies were recorded. Finally, participants were explained about the special design we used. In each condition, a sequence of three consecutive experimental problems appeared with the same symbol (experimental task) followed by a sequence of three control problems cued with “=” symbol (control task). For example, in the choice condition, three problems cued with “?” were alternated with three problems cued with “=.” The two (experimental and control) tasks were separated by a black screen. The duration of the black screen was determined by adding (a) the difference between the maximum duration display of an item and the participant solution latency of each item in a sequence, and (b) 1 s. For instance, suppose participant’s solution latencies were 6.5, 5, and 4 s for the experimental task in the choice condition. The black screen appeared for 6.5 s (i.e., [(7.5 – 6.5) + (7.5 – 5) + (7.5 – 5.5)] + 1 = [1 + 2.5 + 2] + 1). We decided to use block design for two reasons. The first reason was to increase statistical power. Indeed fMRI signal in response to a stimulus is additive. This means that the amplitude of the hemodynamic response increases when multiple stimuli are presented in rapid succession. So block designs offer considerable statistical power in comparison to event-related design because the signal change in the fMRI signal following a single stimulus presentation is small. Thus, block design was favored here in order to maximize brain activations underlying strategy selection. The second reason was to control for the length of each block. We wanted durations to be longer than 12 s so that hemodynamic responses were well stabilized (plateau) for each condition. The goal was to have no bias, due to differences in processing time, across the different conditions.

**FIGURE 1:**
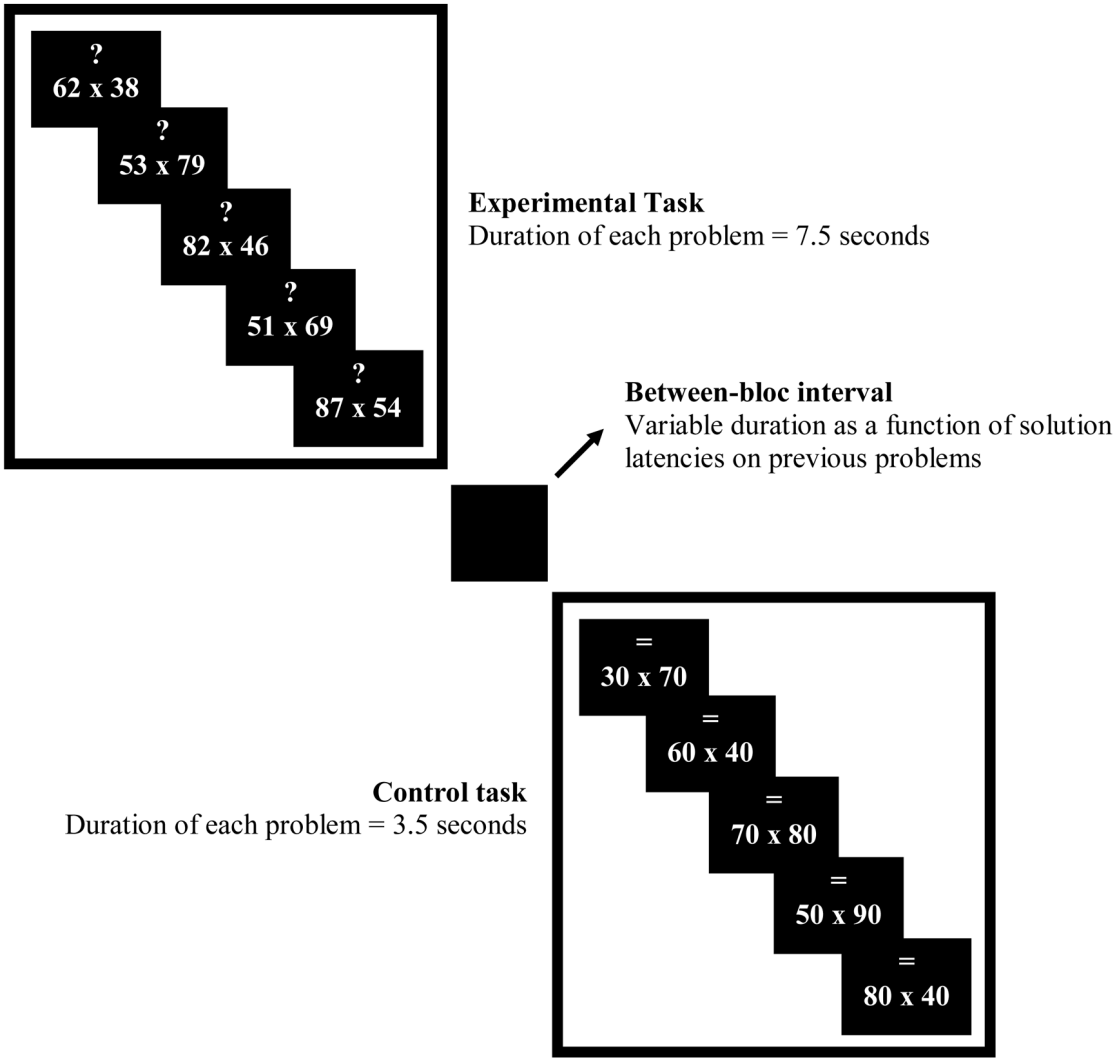
**Experimental design**.

One to 2 weeks later, in the second session, participants were reminded both rounding strategies and were given an additional practice similar to the task to be performed in fMRI scans. The same duration display as in the previous practice was used. However, two parameters differed from the previous practice. First, each sequence included five problems instead of three problems. Second, each set of problems started with an instruction screen which appeared for 14 s. This screen indicated which task to perform in each tested condition (e.g., “?” or “=,” for selection condition; see **Figure 1**).

After a few minutes rest, participants were installed in the scanner. A microphone placed in front of participant’s mouth allowed us to record participant’s response during scanning. Participants underwent four fMRI scans which correspond to two functional selection runs and two functional execution runs. Following Siegler and Lemaire (1997), the choice condition always preceded the no-choice conditions so that strategy selection in the choice condition was not contaminated by recent strategy execution. In each condition, the two functional runs were counterbalanced between participants. Each choice run lasted 12 min and the no-choice runs lasted 7 and 8 min for the RD and the RU strategies, respectively.

### fMRI PROCEDURE

#### Data acquisition

Imaging was performed with a 3T whole-body imager MEDSPEC 30/80 ADVANCE (Bruker). High-resolution structural T1-weighted images were required for all participants to allow precise anatomical localization. The anatomical slices covered the whole brain and were acquired parallel to the anterior–posterior commissure (AC–CP) plane. For each choice run, 342 functional volumes, for RD run 229, and for RU run 244, sensitive to blood oxygen level-dependent (BOLD) contrast were acquired with T2*- weighted echo-planar (EPI) sequence at 32 axial slices (field of view = 192 mm × 192 mm, slice thickness 3 mm; inter-slice gap 1 mm, 64 × 64 matrix of 3 mm × 3 mm voxels). The slices were parallel to AC–CP commissure and covered the whole brain. Acquisition of all functional volumes took place continuously during a single session, with a repetition time (TR) of 2.133 s/volume. The six first volumes were discarded to allow for T1 equilibration effects.

#### Preprocessing

Preprocessing were realized using Statistical Parametric Mapping (SPM8, Wellcome Trust Centre for Neuroimaging, London, UK; Friston et al., 1995, http://www.fil.ion.ucl.ac.uk/spm/software/spm8/). SPM8 software was implemented in MATLAB 7.5 (Mathworks Inc. Sherborn, MA, USA). Because slices were not acquired simultaneously but one after the others, to correct the different volume acquisitions over time, the signal measured in each slice was shifted relative to the acquisition of the middle slice using a tri temporal linear interpolation. To correct movements during volume acquisition, the functional images were realigned on the first image of the first session. For multi-subject analysis, anatomical and realigned functional images of all subjects were transformed (non-linear basis transformations) in a common standard space using the Montréal Neurological Instute (MNI) template. Images were then smoothed with a 6 mm full width half maximum (FWHM) isotropic Gaussian filter to reduce residual anatomical variations between subjects. Finally, data were high-pass filtered with a cut-off period of 128 s to remove from analysis low frequencies corresponding to breathing or heartbeats.

#### Whole brain data analyses

For all participants, activations in each run were modeled using a combination of standard SPM functional hemodynamic response functions. For single-subject analyses, we carried out several analyses. In each run, we contrasted activations on experimental problems minus control problems. First, to delineate strategy selection networks, we contrasted experimental task minus control task in choice condition to experimental task minus control task in no-choice condition. Second, to delineate strategy execution networks, we contrasted experimental task and control task in no-choice RU run to no-choice RD run.

In the second step of statistical analyses, data were submitted to a random-effect group analysis with subjects as a random variable. The contrasts produced statistical parametric maps (SPMs) of the *t* statistics at each voxel, which were subsequently transformed to the unit normal *Z* distribution. Results represent one-sample *t*-tests and paired *t*-tests. For two-samples *t*-tests and two-factorial ANOVAs, threshold for the resulting images was set at *p* < 0.001 level (uncorrected); we kept only the cluster size greater than 20 voxels in order to retain only clusters that passed a corrected threshold (*p* < 0.05).

#### ROIs data analyses

We focused on four important regions following activations found in the whole brain analyses: anterior cingulate cortex (ACC), angular gyrus (ANG), dorso-lateral prefrontal cortex (DLPFC), and SMG. We used the coordinates from the meta-analysis of Arsalidou and Taylor (2011) to locate ACC (*x* = -8, *y* = 8, *z* = 46, and *x* = 4, *y* = 22, *z* = 36), ANG (*x* = -50, *y* = -58, *z* = 42, and *x* = 30, *y* = -58, *z* = 32), SMG (*x* = 54, *y* = -42, *z* = 32, and *x* = -44, *y* = -40, *z* = 42), and the DLPFC (*x* = -30/+30, *y* = 28, *z* = 56) from Pastötter et al.’s (2010) paper. For each ROI, these coordinates represented the center of a 10-mm circle. Then, we applied a mask using anatomical areas available in the Automated Anatomical Labeling (AAL; Tzourio-Mazoyer et al., 2002) to exclude white matter in our ROI. For all participants, activations in each run were modeled using a combination of standard SPM functional hemodynamic response functions.

## RESULTS

### BEHAVIORAL PERFORMANCE

We conducted analyses on mean percentage use of the RD strategy, mean solution times, and mean percentages of errors (i.e., answering 3,100 when rounding down 82 × 47). We removed from the analyses 7.8% of problems on which participants did not click on the button response or clicked too quickly (i.e., solution latencies smaller than 800 ms). In all reported analyses unless otherwise noted, differences are significant to at least *p* < 0.05. Analyses aimed at determining which strategy participants chose to provide the better estimate (choice condition), and how fast and accurate participants executed the required rounding strategy (no-choice condition; see **Table 2**). Two raters who independently transcribed recorded answers agreed on 97% answers.

**Table 2:** Mean solution latencies (in ms) and mean percentages of errors as a function of strategies and problems in each condition.

Conditions	RD strategy			RU strategy			Control
	Small-unit problems	Large-unit problems	Mean	Small-unit problems	Large-unit problems	Mean	
Choice	3716	3847	3782	4680	4452	4566	2333
No-choice	2222	2244	2233	3108	3045	3076	2204
**Percentages of errors**
Choice	3.3	12.5	7.9	8.9	3.9	6.4	5.8
No-choice	1.7	1.1	1.4	2.4	2.0	2.2	2.5

#### Choice condition

Repeated-measure ANOVA with 2 (Problem: small-unit, large-unit problems) on mean percent use of RD strategy revealed a significant effect of problem, *F*(1,17) = 133.8, MSE = 141.55, $\eta_{p}^{2}$ = 0.88, *p* < 0.01. Participants used the RD strategy more often on small-unit (74.7%) than on large-unit problems (28.8%).

Repeated-measure ANOVAs with 2 (Problem: small-unit, large-unit problems) × 2 (Strategy: RD, RU strategies) conducted on mean solution latencies and mean percentages of errors revealed that participants were faster when selecting and executing the RD (3,782 ms) than the RU strategy (4,566 ms), *F*(1,17) = 53.7, MSE = 194790, $\eta_{p}^{2}$ = 0.77, *p* < 0.01. Moreover, the Problem x Strategy interaction, *F*(1,17) = 8.54, MSE = 63932, $\eta_{p}^{2}$ = 0.35, *p* = 0.01, showed that participants were faster when choosing the RU strategy on large-unit problems (4,452 ms) than on small-unit problems (4,680 ms), but equally fast on the two types of problem for the RD strategy (3,847 and 3,716 ms, *F* < 2).

Analyses on mean percentages of errors revealed a significant Problem x Strategy interaction, *F*(1,17) = 4.9, MSE = 904, $\eta_{p}^{2}$ = 0.22, *p* = 0.04, showing that participants made fewer errors when they selected and executed the RD strategy on small-unit problems (3.3% vs. 12.5%, *F* < 4) and the RU strategy on large-unit problems (3.9% vs. 8.9%, *F* < 4) than the reverse.

#### No-choice conditions

We conducted repeated-measure ANOVAs with 2 (Problem: small-unit, large-unit problems) × 2 (Strategy: RD, RU strategies) design on mean solution latencies and on mean percentages of errors. These analyses only revealed a main effect of strategy *F*(1,17) = 242.4, MSE = 52820, $\eta_{p}^{2}$ = 0.93, *p* < 0.01. Participants made fewer errors when executing the RD (1.4%) than the RU strategy (2.2%). No other effects came out significant in either solution latencies or on percent of errors.

### IMAGING DATA

Functional MRI data analyses were carried out to determine the neural bases of strategy selection and of strategy execution. Two main sets of comparisons were conducted. We compared brain activations associated with experimental task relative to control task. First, ANOVA was performed on BOLD signal using condition as factor to identify which regions were associated with strategy selection. Second, paired *t*-tests were used to test specific cerebral regions engaged by RU strategy relative to RD strategy in the no-choice condition. Such method means that all voxels listed as demonstrating significant effects were significant in the choice condition relative to the no-choice condition, and in the no-choice RU condition relative to the no-choice RD condition. We report cerebral activations first for strategy selection and then for strategy execution (see **Table 3**)^1^.

**Table 3:** Brain activations associated with strategy selection and strategy execution.

Size	Peak *Z*	Side	Cerebral region	Tal (*x*)	Tal (*y*)	Tal (*z*)
**Choice > no-choice**
242	5.14	R	Angular gyrus (ANG)	39	-57	42
	5.09	R	Supramarginal gyrus (SMG)	45	-48	48
82	5.26	L	SMG	-36	-45	33
73	3.57	R	Middle frontal gyrus	24	12	45
69	4.46	R	Anterior cingulate cortex (ACC)	6	24	39
58	4.53	R	Precuneus	27	-72	45
42	3.66	L	Middle frontal gyrus	-24	12	51
37	3.99	R	Dorso lateral prefrontal cortex	39	24	45
**RU > RD in no-choice condition**
243	5.24	R	SMG	51	-39	54
	4.97	R	Precuneus	18	-69	51
59	5.78	L	Precuneus	-12	-63	48
42	4.42	R	Middle frontal gyrus	42	3	51
31	4.25	L	Middle frontal gyrus	-21	3	51
23	4.31	L	Middle occipital gyrus	-30	-75	15

*Regions tabulated show significant (*p* < 0.001, cluster size > 20) signal increases for experimental task versus control task. *N* refers to the number of significant voxels in each cluster, *Z* to the *Z* statistic value for each peak, and *x*, *y*, *z* refer to the distances in mm from the origin in Talairach space.*

#### Neural network involved in the choice condition

**FIGURE 2:**
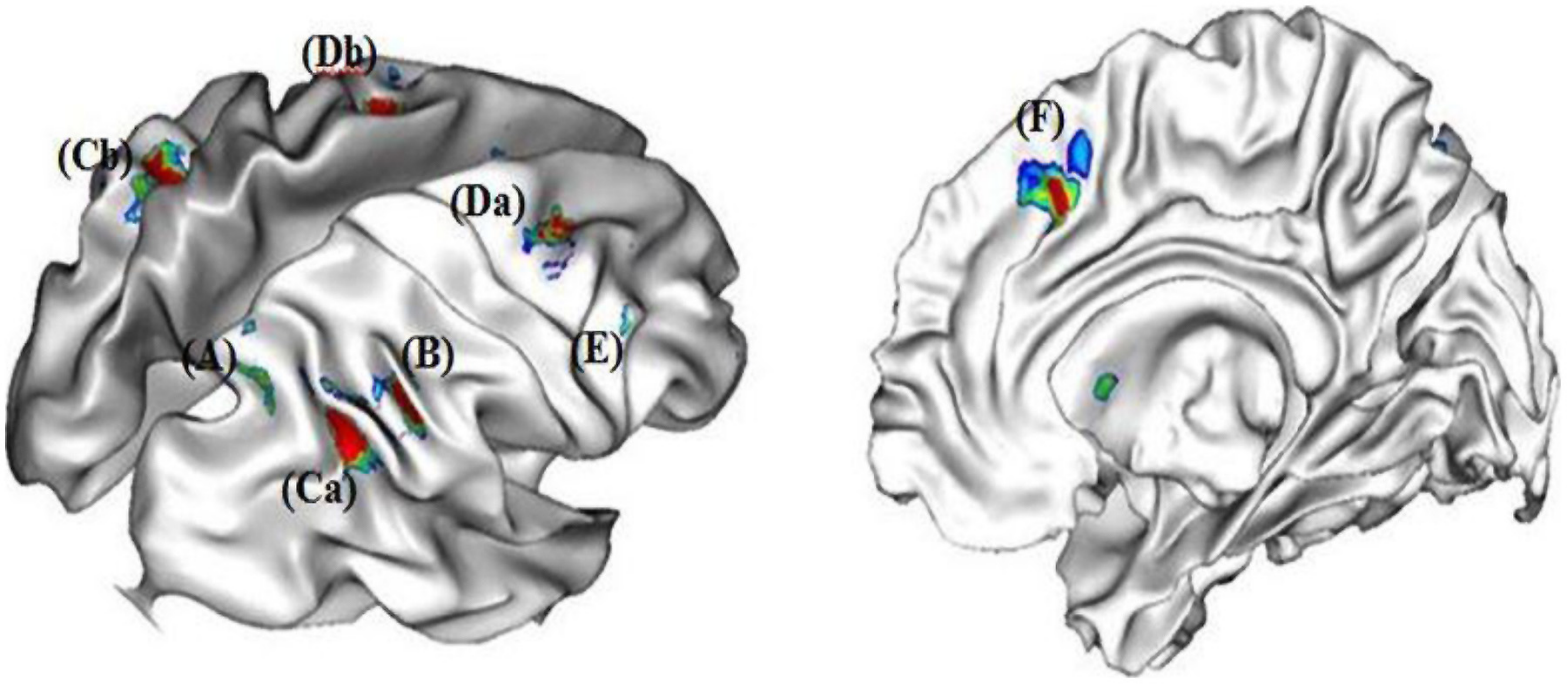
**Neural correlates of strategy selection (A) right precuneus, (B) right angular gyrus, (Ca) right and (Cb) left supramarginal gryi, (Da) right and (Db) left middle frontal gyri, (E) right dorso-lateral prefrontal cortex (DLPFC), and (F) right anterior cingulated cortex**.

In our experiment, strategy selection required participants to choose between two rounding strategies and to execute the chosen strategy. The contrast between the choice condition and the no-choice condition revealed specific activations in inferior parietal cortex (i.e., right and left SMG, right ANG, and right precuneus), frontal cortex (right DLPFC, and right and left middle frontal gyrus) and ACC (see **Figure 2**). As selecting a strategy involved to execute this strategy, the no-choice/choice contrast revealed no specific activations.

#### Neural network involved in the no-choice RU condition

**FIGURE 3:**
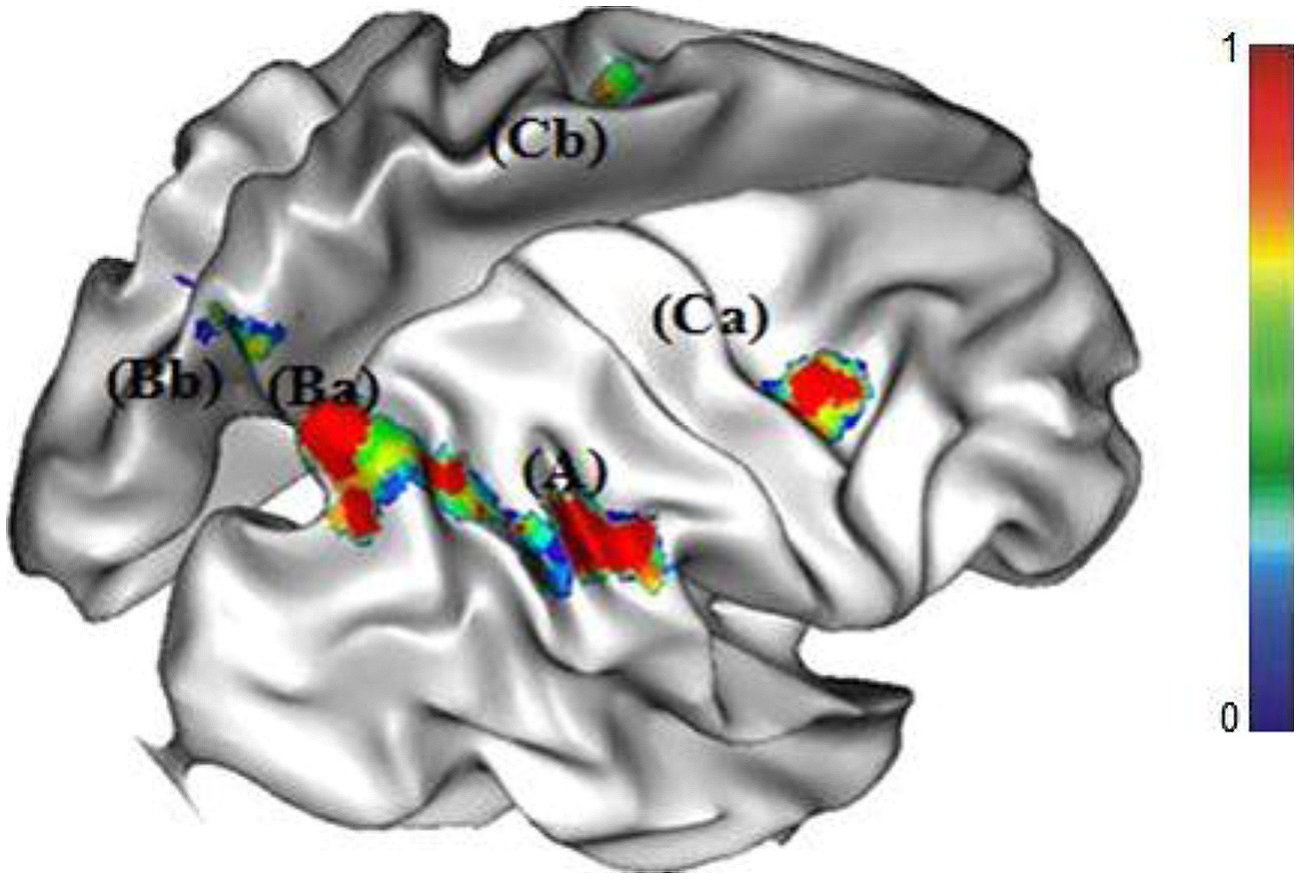
**Cortical regions of increased activations for the RU strategy (A) right supramarginal gyrus (SMG), (Ba) right and (Bb) left precuneus, (Ca) right and (Cb) left middle frontal gyri**.

The comparison between the no-choice RU and the no-choice RD conditions revealed a specific cerebral network involving activations in parietal cortex (i.e., right and left precuneus, right SMG), right and left middle frontal gyri, and left middle occipital gyrus (see **Figure 3**).

The absence of brain activations in contrasting the no-choice RD and the no-choice RU conditions is consistent with the fact that the RU strategy involved incrementing each decade relative to the RD strategy. Moreover, as control task is highly similar to RD strategy execution, no clusters passed through the threshold of 20 voxels when comparing the experimental task to the control task in the no-choice RD condition.

#### ROIs analyses

The GLM yielded four beta weights per ROI for each participant. A two-way ANOVA was performed, using the mean activation values in each ROI, with a within-subjects factor of 2 (Condition: choice, no-choice) × 4 (ROI: ACC, DLPFC, ANG, SMG) design. Analysis revealed significant effects of Condition *F*(1,17) = 5.68, MSE = 2.66, $\eta_{p}^{2}$ = 0.25, *p* = 0.03, showing less activations in the no-choice than in the choice condition. We also found a Condition × ROI interaction, *F*(1,17) = 3.68, MSE = 0.34, $\eta_{p}^{2}$ = 0.18, *p* = 0.02, showing greater activation, for choice than no-choice condition, in ACC, *F*(1,17) = 5.44, MSE = 1.76, $\eta_{p}^{2}$ = 0.24, *p* = 0.03, than in ANG, *F*(1,17) = 7.44, MSE = 0.63, $\eta_{p}^{2}$ = 0.30, *p* = 0.01, than in DLPFC, *F*(1,17) = 6.93, MSE = 0.66, $\eta_{p}^{2}$ = 0.29, *p* = 0.02, but not in SMG, *F* < 1, *p* = 0.63.

## DISCUSSION

In this study, we investigated neural correlates of strategy selection and strategy execution. In the context of an arithmetic computational estimation task, participants were asked to select the better strategy in a choice condition, and to execute a pre-determined strategy on each problem in no-choice conditions. We replicated previously found behavioral data regarding strategy selection and strategy execution. Indeed, participants performed the strategy selection task as expected as they chose more often than chance the better strategy on each problem. In the execution task, the RD strategy was faster than the RU strategy. These results suggest that participants accomplished our computational estimation task in the scanner the same way as outside the scanner. Most originally, the present data revealed specific brain activations involving (1) ACC, DLPFC, and ANG for strategy selection, and (2) occipito-parietal networks for strategy execution processes. We discuss implications of these findings for further our understanding of strategic aspects of human cognition.

### STRATEGY SELECTION NETWORK

The contrast between activations in the choice and in the no-choice conditions revealed specific activations in the right hemisphere including ACC, DLPFC, ANG, and bilateral activations in SMG and in middle frontal gyrus.

Regarding the choice condition, it is important to emphasize that to obtain further evidence that participants were not randomly choosing the better strategy, we collected participants’ verbal reports outside the scanner. All participants reported that they added the two unit digits and compared the sum of unit digits to a reference number (i.e., 10) to decide which strategy was the better strategy. Participants chose RU when the sum of unit digits was larger than or equal to 10, and decided to round down when the sum of unit digits was smaller or equal to 10. It is possible that activations in the right ANG reflect the focused attention on the unit digits and the comparison to the reference number 10. Göbel et al. (2001) investigated the role of ANG in a Transcranial Magnetic Stimulation (TMS) study to understand the function of this brain region. They contrasted performance in a visuo-spatial task (i.e., deciding of the presence or absence of a specific stimulus among distractor stimuli) and a number comparison task (i.e., deciding whether a two-digit number is smaller or larger than 65), while disrupting activations in ANG. They found that number processing was greatly impaired with TMS in the left ANG whereas deficits in visuo-spatial task were more important after TMS stimulation in the right ANG. Such findings are consistent with well-known literature on right hemisphere’s activations for visuo-spatial attention (e.g., Corbetta and Shulman, 1999) and number processing activations associated with left hemisphere (e.g., Dehaene, 1999). In our study, participants had to decide which cue is important to choose the better strategy in the choice condition. Using the sum of unit digits heuristic to do so, the task goal representation (i.e., choosing the better strategy) could have been more precisely defined as first calculating the sum of unit digits, comparing this sum to 10, to decide to use RU or RD. Such an interpretation is also consistent with the role of ANG in tasks involving goal-directed salience representations (Zenon et al., 2010), and facts retrieval in long-term memory during problem solving (Menon et al., 2000; Grabner et al., 2009).

In parietal cortex, we expected greater activations in SMG as participants had to maintain an intermediate result as a verbal storage (e.g., Smith and Jonides, 1998). In our study, intermediate step may consist in maintaining active the sum of unit digits and both strategies in memory during processing of unit digits. Participants may maintain strategies as verbal storage as “Down” and “Up” strategies linked to strategy procedures (i.e., multiplying the decades for RD, and increasing the decades and multiplying them for RU). However, even if in the no-choice condition, a pre-determined strategy had to be executed, participants may maintain these rounding procedures as verbal storage, leading to no specific activations in SMG when contrasting the choice and no-choice conditions.

As sum of unit digits and strategy procedures needed to be actively maintained, activations in DLPFC are consistent with maintenance in working memory, selection processes (Curtis and D’Esposito, 2003, for a review), and cognitive control (e.g., Koechlin et al., 2003; Badre and Wagner, 2004; Ridderinkhof et al., 2004; Braver and Barch, 2006). Badre and Wagner (2004) investigated prefrontal cognitive control mechanisms while focusing on the nature of DLPFC, frontopolar cortex (FPC), and ACC structures. Brain activations in DLPFC were mostly associated with tasks involving working memory (e.g., Petrides, 2000; Curtis and D’Esposito, 2003), strategic organization during encoding (e.g., Shimamura, 1995; Blumenfeld and Ranganath, 2006, 2007), and response selection (e.g., MacDonald et al., 2000; Rowe et al., 2000; Miller and Cohen, 2001). In our experiment, in addition to select relevant information, activations in the DLPFC may be linked to an updating of relevant information (i.e., small or large unit digits) leading to inhibiting one of the two rounding strategies.

Anterior cingulate cortex activations found here are consistent with studies in decision making (e.g., Walton et al., 2007; Anderson et al., 2009a; D’Esposito et al., 2009; Boorman et al., 2011; Rushworth et al., 2011). Anderson et al. (2009a) compared brain activations predicted by their ACT-R model (Adaptive Control of Thoughts, Lovett and Anderson, 1996) to brain activations when participants solve arithmetic equations (e.g., *7x + 3 = 38)*. When participants decided to solve *+3* before solving *7x*, ACC was activated as predicted by the model. These activations in ACC were interpreted as sustaining which decision to make after encoding equation information. In our study, greater activations in ACC may reflect strategy selection and regulation processes. That is, after choosing and executing a strategy, the just executed strategy efficiency may be associated with the relevant cue, the sum of unit digits. Such interpretation is supported by activations in ACC found to support regulation processes (MacDonald et al., 2000; Sohn et al., 2007).

Investigating more precisely PFC, Koechlin et al. (2003) proposed a cascade model of cognitive control organized in the lateral PFC following a rostrocaudal axis (see also Badre, 2008). The functional model showed that sensory (i.e., motor response), contextual (i.e., selecting the appropriate response according to the stimulus) and episodic control (i.e., relevant past events) were implemented in premotor, caudal, and rostral lateral PFC regions, respectively. Koechlin and Summerfield (2007) specified that anterior DLPFC activations were associated with maintaining or monitoring the rules for action, while activity in posterior DLPFC varied with the contexts (i.e., contextual signals guide response selection). Posterior regions of PFC were shown to subserve selection mechanisms. Furthermore, Koechlin and Hyafil (2007) described a neurocomputational model of how a reward task set is placed from an ongoing state to a pending state, and reverting back to an active state later, in anterior PFC. The authors showed that FPC implemented reward-based cognitive branching in interaction with anterior lateral and medial/orbital prefrontal regions. However, the model was limited to two identical expected reward task sets, suggesting that complex decision making may rely on other brain structures like parietal cortex which supported formation of mental spatial maps of multiple branching sets. This is consistent with activation in PFC and parietal areas in our study.

Finally, studies on working memory found cerebral activity in a fronto-parietal network (e.g., Brass and von Cramon, 2004; Roth et al., 2006) similar to the network found in task switching studies (e.g., Dosenbach et al., 2006; Slagter et al., 2006). Montojo and Courtney (2008) specified that in the fronto-parietal update network, inferior frontal junction exhibited greater activation for rule update whereas intraparietal sulcus showed greater activation for stimulus update. Also, the fronto-parietal network is recruited for top–down attention control (Knight et al., 1995; Miller, 2000; Knight, 2007). Wang et al. (2010) suggested that in such network, ACC and DLPFC interact with each other to resolve conflict, and intraparietal sulcus was associated with attention control.

Our findings have important theoretical implications as brain activations found here bring further evidence for the involvement of executive functions in strategy selection. Some models, like ACT-R and SSL, argue that there are close relations between strategy selection and activated associations from long-term memory. However, this does not exclude executive functions during strategy selection. Thus, involvement of executive functions in the strategy selection is not incompatible with models of strategy selection involving considerations of strategy costs/benefits if an evaluation of relative costs and benefits of different strategies requires controlled attentional resources. Nevertheless, current strategy selection models do not explicitly assume any role of executive functions, it would be interesting to test whether implementing executive functions in these computational models would account for age-related differences in strategic variations (e.g., in arithmetic, Hodzik and Lemaire, 2011; in memory, Taconnat et al., 2009). Executive functions may be involved in maintaining active strategy procedures or focused attention, updating relevant information, and/or inhibiting irrelevant information when choosing a strategy among several ones, processes that could easily be implemented in current models of strategy selection.

### STRATEGY EXECUTION NETWORKS

The networks associated with execution of RU showed activations in parietal cortex (i.e., right and left precuneus, right SMG), right and left middle frontal gyri, and left middle occipital gyrus.

Such activations are consistent with activations reported by Zago et al. (2001) when they contrasted activations of a *compute condition* to a *read condition*. In their *compute condition*, participants solved two-by-two digit number multiplication problems (24 × 13). To solve such multiplication problems, participants used an algorithm involving several steps which required retrieving intermediate results in memory. In their *read condition*, participants saw pairs of Arabic digits composed of zero and one (e.g., 1 0) and had to read them aloud (e.g., “one zero”). The authors found activations in middle frontal gyrus associated with working memory tasks and executive processes (e.g., Courtney et al., 1998; Smith and Jonides, 1998, 1999) and with simple multiplication problems solving task (e.g., Chochon et al., 1999; Pesenti et al., 2001).

In our study, right SMG activations may be related to active working-memory maintenance of the increased decades. This interpretation is supported by studies showing activations in this brain area when intermediate results are maintained as verbal storage (e.g., Smith and Jonides, 1998).

Activities in middle occipital gyri were found when complex stimuli were presented (e.g., multi-digits vs. single-digit visual processing; Dehaene and Cohen, 1997). Such activations can be interpreted as manipulating larger numbers when executing RU as compared to RD.

Furthermore, to round up, participants may focus their attention on each decade to increment each of them and shift their attention from the decade localization to another so as to avoid the interference between the incremented decades and presented decades. This interpretation is consistent with precuneus activations found to exhibit activations when participants voluntarily shift attention in different visuo-spatial tasks (Simon et al., 2002) and in tasks requiring spatial information (see Cavanna and Trimble, 2006, for an overview of behavioral correlates of precuneus). Bilateral activations in precuneus may support more focused and shifts of attention for RU than for RD.

In conclusion, this study provided evidence that cognitive demands of each strategy can be seen in both behavioral and neural differences between RU and RD. Each rounding strategy here was associated with specific neural network activations. Moreover, strategy selection was also interestingly associated with specific brain activations. These brain activations for strategy selection overlap in structures known to be activated in decision making. Results showed that DLPFC and ACC might play a crucial role in strategy selection. Functionally, this supports that strategy selection involves executive control processes. As these processes were not included in theoretical models of strategy selection, our findings suggest revisions of these models to explicitly take into account their role in explaining how participants try to choose the better strategy on each item while accomplishing cognitive tasks.

## Conflict of Interest Statement

The authors declare that the research was conducted in the absence of any commercial or financial relationships that could be construed as a potential conflict of interest.

## Footnotes

1 The same analyses were conducted with motion parameters of participants as a covariate. The same results were found.
